# Supplementary material for: Weathering of Antibacterial Melt-Spun Polyfilaments Modified by Pine Rosin
Source: Molecules. 2021 Feb 7;26(4):876. doi: 10.3390/molecules26040876 (PMC7916070; doi:10.3390/molecules26040876)
Supplement: Supplementary file 1 [file molecules-26-00876-s001.pdf]

SUPPLEMENTARY DATA (non-published material)

FOR (title):

Weathering and safety of antibacterial polymer-rosin polyfilaments

by

Mikko Kanerva, Jacob Mensah-Attipoe, Arja Puolakka, T.M. Takala, Marko Hyttinen,

Rama Layek, Sarianna Palola, Vladimir Yudin, Pertti Pasanen, Per Saris

submitted 16.11.2020 peer review

Data description

This data is a detail report related to the VOC measurements described and analyzed in the paper **Weathering and safety of antibacterial polymer-rosin polyfilaments.** The detailed description of the method, samples preparations and main outcomes are given in the manuscript. Tables 1-7 indicate the average and (range) concentration of emitted compounds from each polyfilament fibre sample analyzed at different temperatures.

Table 1. Average and (range) concentration of emitted compounds from fPE analyzed at different temperatures.

	**Average and (range) of concentrations (ng/l/g) of compounds emitted from PE fibres tested at different temperatures**		
Component	**25 ˚C**	**60 ˚C**	**105 ˚C**
1,4-Methanoazulene, decahydro-4,8,8-trimethyl-9-methylene-, [1S-(1.alpha.,3a.beta.,4.alpha.,8a.beta.)]-	93 (77-108)	2236 (1752-2721)	26676 (23389 - 29963)
Tetradecane	5 (0-5)	2386 (1818-2954)	72882 (64173-81590)
Dodecane	18 (10-27)	1555 (972-2139)	29482 (24571-34393)
Bicyclo[2.2.1]heptan-2-ol, 1,7,7-trimethyl-, (1S-endo)-		12 (0-12)	99 (81-118)
Tricyclo[5.4.0.0(2,8)]undec-9-ene, 2,6,6,9-tetramethyl-	5 (3-6)	136 (100-173)	1485 (1223-1747)
Decane		123(73-173)	3908 (3533-4283)
Naphthalene, 1,2,3,4-tetrahydro-1,6-dimethyl-4-(1-methylethyl)-, (1S-cis)-		158 (127-190)	2411 (2013-2808)
Bicyclo[2.2.1]heptan-2-ol, 1,7,7-trimethyl-, acetate, (1S-endo)-		59(39-80)	432(331-522)
.alpha.-Cubebene	10 (0-10)	38 (25-51)	3665 (2885-4444)
2-Oxabicyclo[2.2.2]octan-6-ol, 1,3,3-trimethyl-		15 (0-15)	
1-Butanol	154 (0-154)	210 (0-210)	87(0-87)
Caryophyllene	6 (4-8)	185 (121-249)	2062 (1564-2561)
Decanal		68 (54-82)	717 (491-942)
Nonanal	8 (0-8)	96 (55-136)	624 (402-845)
1,4-Methano-1H-indene, octahydro-4-methyl-8-methylene-7-(1-methylethyl)-, [1S-(1.alpha.,3a.beta.,4.alpha.,7.alpha.,7a.beta.)]-		59 (44-74)	709 (571-848)
Bicyclo[2.2.1]heptan-2-ol, 1,3,3-trimethyl-		7 (6-8)	36 (0-36)
Glycerin			2027 (1620-2434)
Naphthalene, 1,2,4a,5,6,8a-hexahydro-4,7-dimethyl-1-(1-methylethyl)-, (1.alpha.,4a.alpha.,8a.alpha.)-		67 (0-67)	1530 (1430-1629)
Copaene		248 (175-320)	
Dodecane, 2,6,11-trimethyl-			790 (0-790)
Pentadecane		17 (13-21)	647 (446-847)

Nonane, 2,6-dimethyl-			188 (171-204)
Naphthalene, 1,2,4a,5,6,8a-hexahydro-4,7-dimethyl-1-(1-methylethyl)-		20 (18-22)	699 (364-1034)
Tridecane	8 (0-8)	23 (16-29)	458(527-389)
Naphthalene, 1,2,3,4,4a,5,6,8a-octahydro-7-methyl-4-methylene-1-(1-methylethyl)-, (1.alpha.,4a.alpha.,8a.alpha.)-		98 (39-157)	45 4(52-855)

Table 2. Average and (range) concentration of emitted compounds from fPE10 analyzed at different temperatures.

	**Average and (range) of concentrations (ng/l/g) of compounds emitted from PE-rosin fibres tested at different temperatures**		
Component	**25 ˚C**	**60 ˚C**	**105 ˚C**
1,4-Methanoazulene, decahydro-4,8,8-trimethyl-9-methylene-, [1S-(1.alpha.,3a.beta.,4.alpha.,8a.beta.)]-	66 (50-83)	2450 (2002-2898)	85213 (59066-111359)
Tetradecane		1011 (919-1104)	37949 (32830-43068)
Dodecane	8 (7-10)	633 (618-648)	19102 (14329-23875)
Propanoic acid, 2-methyl-		138 (70-206)	8141 (4247-12035)
Bicyclo[2.2.1]heptan-2-ol, 1,7,7-trimethyl-, (1S-endo)-		71 (60-83)	5989 (5540-6438
Tricyclo[5.4.0.0(2,8)]undec-9-ene, 2,6,6,9-tetramethyl-		101 (75-127)	3234 (1997-4472)
Decane		11 (10-12)	745 (666-823)
Naphthalene, 1,2,3,4-tetrahydro-1,6-dimethyl-4-(1-methylethyl)-, (1S-cis)-		96 (84-107)	2042 (1669-2416)
Bicyclo[2.2.1]heptan-2-ol, 1,7,7-trimethyl-, acetate, (1S-endo)-		100 (0-100)	3768 (2781-4755)
.alpha.-Cubebene		14 (12-16)	433 (345-521)
2-Oxabicyclo[2.2.2]octan-6-ol, 1,3,3-trimethyl-		98 (0-98)	4045 (3069-5020)
1-Butanol	21(8-35)	594 (410-777)	2699 (2217-3181)
1,2,4-Methenoazulene, decahydro-1,5,5,8a-tetramethyl-, [1S-(1.alpha.,2.alpha.,3a.beta.,4.alpha.,8a.beta.,9R*)]-		68(0-68)	3232 (1175-5290)
Caryophyllene		25(0-25)	615 (602-628
Decanal	6 (0-6)	66(60-73)	1410 (1094-1727)
Nonanal	9 (0-9)	109(97-121)	1403 (1369-1437)
1,4-Methano-1H-indene, octahydro-4-methyl-8-methylene-7-(1-methylethyl)-, [1S-(1.alpha.,3a.beta.,4.alpha.,7.alpha.,7a.beta.)]-		45(36-55)	1357 (1061-1653)
Benzenemethanol, .alpha.,.alpha.,4-trimethyl-		55(53-57)	2082 (1667-2497)
Bicyclo[2.2.1]heptan-2-ol, 1,3,3-trimethyl-		26(17-35)	2062 (1726-2397)
Naphthalene, 1,2,4a,5,6,8a-hexahydro-4,7-dimethyl-1-(1-methylethyl)-, (1.alpha.,4a.alpha.,8a.alpha.)-		46(0-46)	
Copaene		102(97-107)	2186 (0-2186)
Dodecane, 2,6,11-trimethyl-			1041 (1012-1070
Pentadecane		18(0-18)	621 (595-648)
Bicyclo[2.2.1]heptan-2-one, 1,7,7-trimethyl-, (1R)-			1090 (755-1424)
Nonane, 2,6-dimethyl-		65 (0-65)	828 (585-1071)

Naphthalene, 1,2,4a,5,6,8a-hexahydro-4,7-dimethyl-1-(1-methylethyl)-		9 (0-9)	544 (0-544)
Tridecane		19 (17-21)	491 (430-552)
Hexanoic acid		8 (0-8)	952 (682-1223
Naphthalene, 1,2,3,4,4a,5,6,8a-octahydro-7-methyl-4-methylene-1-(1-methylethyl)-, (1.alpha.,4a.alpha.,8a.alpha.)-		28 (26-30)	662 (0-662)
Phenol, 2,5-bis(1,1-dimethylethyl)-			1780 (0-1780)

Table 3. Average and (range) concentration of emitted compounds from fPA analyzed at different temperatures.

	**Average and (range) of concentrations (ng/l/g) of compounds emitted from PA fibres tested at different temperatures**		
Component	**25 ˚C**	**60 ˚C**	**105 ˚C**
Caprolactam		1028 (859-1198)	55602 (41137-70066)
1,4-Methanoazulene, decahydro-4,8,8-trimethyl-9-methylene-, [1S-(1.alpha.,3a.beta.,4.alpha.,8a.beta.)]-	3 (0-3)	15 (6-25)	125 (64-187)
Caryophyllene			49 (25-73)
1-Butanol	30 (25-34	594 (448-739)	778 (298-1258)
Naphthalene, 1,2,3,4-tetrahydro-1,6-dimethyl-4-(1-methylethyl)-, (1S-cis)-		6 (0-6)	16 (0-16)
Naphthalene, 1,2,3,5,6,8a-hexahydro-4,7-dimethyl-1-(1-methylethyl)-, (1S-cis)-		2 (0-2)	7 (0-7)
Decanal	26 (0-26)	37 (29-42)	298 (0-298)
Naphthalene, 1,2,4a,5,6,8a-hexahydro-4,7-dimethyl-1-(1-methylethyl)-, (1.alpha.,4a.alpha.,8a.alpha.)-			9 (0-9)
Tetradecane	6 (0-6)	40 (10-70)	148 (143-152)
Nonanal		30 (23-36)	139 (43-234)
Acetic acid			1656 (0-1656)
Cyclotrisiloxane, hexamethyl-	149 (3-296)	56 (49-63)	128 (113-142)
Bicyclo[2.2.1]heptan-2-ol, 1,7,7-trimethyl-, (1S-endo)-		4 (0-4)	
Naphthalene, 1,2,3,4,4a,5,6,8a-octahydro-7-methyl-4-methylene-1-(1-methylethyl)-, (1.alpha.,4a.alpha.,8a.alpha.)-			3 (0-3)
Benzene, 1-methyl-2-(1-methylethyl)-		14 (0-14)	
Benzyl Alcohol		15 (0-15)	205 (97-313)
Cyclotetrasiloxane, octamethyl-	107 (0-107)	23 (21-24)	49 (35-63)

Table 4. Average and (range) concentration of emitted compounds from fPA10 analyzed at different temperatures.

	**Average and (range) of concentrations (ng/l/g) of compounds emitted from PA-rosin fibres tested at different temperatures**		
Component	**25 ˚C**	**60 ˚C**	**105 ˚C**
Caprolactam		473 (376-570)	81710 (78497-84923)

1,4-Methanoazulene, decahydro-4,8,8-trimethyl-9-methylene-, [1S-(1.alpha.,3a.beta.,4.alpha.,8a.beta.)]-		58 (18-98)	15282 (8246-22318)
Caryophyllene		14 (0-14)	10273 (5725-14820)
p-menth-1-en-8-ol			8267 (5473-11061)
1-Butanol	158 (0-158)	337 (0-337	958(700-1217)
Copaene		11 (0-11)	2219 (1271-3168)
Naphthalene, 1,2,3,4-tetrahydro-1,6-dimethyl-4-(1-methylethyl)-, (1S-cis)-		13 (0-13)	2083 (1170-2995)
Naphthalene, 1,2,3,5,6,8a-hexahydro-4,7-dimethyl-1-(1-methylethyl)-, (1S-cis)-		3 (0-3)	2058 (887-3229)
.alpha.-Caryophyllene			1759 (1036-2481)
Bicyclo[2.2.1]heptan-2-ol, 1,7,7-trimethyl-, acetate, (1S-endo)-			1333 (925-1741)
Decanal	9 (0-9)	46 (44-49)	838 (472-1203)
D-Limonene			1043 (675-1412)
Tricyclo[5.4.0.0(2,8)]undec-9-ene, 2,6,6,9-tetramethyl-			1014 (573-1455)
Naphthalene, 1,2,4a,5,6,8a-hexahydro-4,7-dimethyl-1-(1-methylethyl)-, (1.alpha.,4a.alpha.,8a.alpha.)-		4 (0-4)	903 (483-1323)
1R-.alpha.-Pinene			887 (576-1197)
Tetradecane		95 (25-165)	586 (246-926)
Nonanal		36 (34-38)	664 (470-858)
Propanoic acid, 2-methyl-			825 (629-1021)
Pentanamide			811 (647-976)
Cyclotrisiloxane, hexamethyl-	6 (0-6)	22 (14-29)	442 (313-571)
Bicyclo[2.2.1]heptan-2-ol, 1,7,7-trimethyl-, (1S-endo)-			766 (542-991)
Naphthalene, 1,2,3,4,4a,5,6,8a-octahydro-7-methyl-4-methylene-1-(1-methylethyl)-, (1.alpha.,4a.alpha.,8a.alpha.)-			651 (340-962)
Bicyclo[2.2.1]heptan-2-ol, 1,3,3-trimethyl-			531 (354-707)
Benzenemethanol, .alpha.,.alpha.,4-trimethyl-			519 (362-677)
1,4-Methano-1H-indene, octahydro-4-methyl-8-methylene-7-(1-methylethyl)-, [1S-(1.alpha.,3a.beta.,4.alpha.,7.alpha.,7a.beta.)]-			508 (318-697)
Benzene, 1-methyl-2-(1-methylethyl)-			898 (0-898)
2-Oxabicyclo[2.2.2]octan-6-ol, 1,3,3-trimethyl-			430 (302-559)
Bicyclo[3.1.1]heptane, 6,6-dimethyl-2-methylene-, (1S)-			814 (0-814)
Benzyl Alcohol		7 (0-7)	189 (183-195)
Cyclotetrasiloxane, octamethyl-	5 (4-7)	11 (6-17)	240 (169-311)

Table 5. Average and (range) concentration of emitted compounds from fPLA analyzed at different temperatures.

	**Average and (range) of concentrations (ng/l/g) of compounds emitted from PLA fibres tested at different temperatures**	
Component	**60 °C**	**105 °C**
Caryophyllene		119 (41-196)

1,4-Methanoazulene, decahydro-4,8,8-trimethyl-9-methylene-, [1S-(1.alpha.,3a.beta.,4.alpha.,8a.beta.)]-	56 (15-97)	776(75-1477)
1,4-Dioxane-2,5-dione, 3,6-dimethyl-, (3S-cis)-	33 (31-35	13716 (12301-15131)
.alpha.-Caryophyllene		29(0-29)
Bicyclo[3.1.1]heptane, 6,6-dimethyl-2-methylene-, (1S)-		31(0-31)
Tricyclo[5.4.0.0(2,8)]undec-9-ene, 2,6,6,9-tetramethyl-		51(0-51)
Naphthalene, 1,2,3,5,6,8a-hexahydro-4,7-dimethyl-1-(1-methylethyl)-, (1S-cis)-	10 (0-10)	29(0-29)
.alpha.-Cubebene		86(0-86)
Tetradecane	103 (11-195)	403 (59-747)
1-Butanol	282 (120-444)	962 (898-1027)
Dodecane	36 (0-36)	197 (53-341)
Propanoic acid, 2-methyl-		56 (0-56)
Benzene, 1-methyl-4-(1-methylethyl)-		24 (0-24)
Benzenemethanol, .alpha.,.alpha.,4-trimethyl-	6 (6-7)	
Naphthalene, 1,2,3,4,4a,5,6,8a-octahydro-7-methyl-4-methylene-1-(1-methylethyl)-, (1.alpha.,4a.alpha.,8a.alpha.)-		21(0-21)

Table 6. Average and (range) concentration of emitted compounds from fPLA1Ag analyzed at different temperatures.

	**Average and (range) of concentrations (ng/l/g) of compounds emitted from PLA-Ag fibres tested at different temperatures**	
Component	**60 °C**	**105 °C**
1,4-Methanoazulene, decahydro-4,8,8-trimethyl-9-methylene-, [1S-(1.alpha.,3a.beta.,4.alpha.,8a.beta.)]-		133 (0-133)
1,4-Dioxane-2,5-dione, 3,6-dimethyl-, (3S-cis)-	86 (71-101)	24155 (23386-24925)
Tetradecane	145 (79-211)	3654 (1908-5399)
1-Butanol	646 (147-1145)	1835 (854-2816)
Dodecane	51 (0-51)	2937 (1615-4259)
2-Pyrrolidinone		3030 (2792-3268)

Table 7. Average and (range) concentration of emitted compounds from fPLA10 analyzed at different temperatures.

	**Average and (range) of concentrations (ng/l/g) of compounds emitted from PLA-rosin fibres tested at different temperatures**	
Component	**60 °C**	**105 °C**
Caryophyllene	90 (73-107)	215914 (136140-295689)
1,4-Methanoazulene, decahydro-4,8,8-trimethyl-9-methylene-, [1S-(1.alpha.,3a.beta.,4.alpha.,8a.beta.)]-	83 (67-99)	193568 (118229-268908)
p-menth-1-en-8-ol		82381 (48045-116717)
Copaene	12 (9-15)	43724 (23711-63736)
1,4-Dioxane-2,5-dione, 3,6-dimethyl-, (3S-cis)-		6055 (0-6055)

.alpha.-Caryophyllene		36128 (18660-53596)
1S-.alpha.-Pinene	5 (0-5)	35163 (24633-45693)
Naphthalene, 1,2,4a,5,8,8a-hexahydro-4,7-dimethyl-1-(1-methylethyl)-, [1S-(1.alpha.,4a.beta.,8a.alpha.)]-	21 (0-21)	64868 (0-64868)
Bicyclo[3.1.1]heptane, 6,6-dimethyl-2-methylene-, (1S)-		29697 (19962-39433)
D-Limonene		23506 (15839-31173)
Tricyclo[5.4.0.0(2,8)]undec-9-ene, 2,6,6,9-tetramethyl-	6 (5-7)	23125 (12333-33918)
Naphthalene, 1,2,3,5,6,8a-hexahydro-4,7-dimethyl-1-(1-methylethyl)-, (1S-cis)-	24 (0-24)	20411 (17235-23588)
Cyclohexene, 1-methyl-4-(1-methylethylidene)-	10 (0-10)	19106 (12670-25542)
.alpha.-Cubebene		15565 (7268-23862)
Acetic acid		12781 (9427-16136)
Bicyclo[2.2.1]heptan-2-ol, 1,7,7-trimethyl-, acetate, (1S-endo)-		11711 (6206-17216)
Naphthalene, 1,2,4a,5,6,8a-hexahydro-4,7-dimethyl-1-(1-methylethyl)-, (1.alpha.,4a.alpha.,8a.alpha.)-	4 (0-4)	9640 (5227-14053)
Naphthalene, 1,2,4a,5,6,8a-hexahydro-4,7-dimethyl-1-(1-methylethyl)-		8279 (2232-14326)
1,4-Methano-1H-indene, octahydro-4-methyl-8-methylene-7-(1-methylethyl)-, [1S-(1.alpha.,3a.beta.,4.alpha.,7.alpha.,7a.beta.)]-		6582 (3565-9598)
Bicyclo[2.2.1]heptan-2-ol, 1,3,3-trimethyl-		5871 (3264-8477)
Toluene		5175 (4433-5917)
Ylangene		4447 (2461-6432)
3-Isopropylbenzaldehyde	14 (0-14)	4399 (2504-6293)
Tetradecane	14 (13-16)	
1-Butanol	514 (100-928)	
Borneol		8408 (0-8408)
3-Cyclohexen-1-ol, 4-methyl-1-(1-methylethyl)-, (R)-		3740 (2140-5340)
Bicyclo[3.1.0]hex-3-en-2-ol, 2-methyl-5-(1-methylethyl)-, (1.alpha.,2.alpha.,5.alpha.)-		3722 (2557-4887)
Bicyclo[3.1.1]heptan-3-ol, 6,6-dimethyl-2-methylene-, [1S-(1.alpha.,3.alpha.,5.alpha.)]-		3334 (1833-4834)
(+)-Epi-bicyclosesquiphellandrene		6390 (0-6390)
Dodecane		
Cyclohexene, 6-ethenyl-6-methyl-1-(1-methylethyl)-3-(1-methylethylidene)-, (S)-		3065(1873-4257)
Propanoic acid, 2-methyl-	11 (0-11)	2999(1358-4639)
Benzene, 1-methyl-4-(1-methylethyl)-	5 (0-5)	2691(224-5157)
Benzenemethanol, .alpha.,.alpha.,4-trimethyl-	7 (0-7)	2591(1316-3866)
1H-Cyclopropa[a]naphthalene, 1a,2,3,5,6,7,7a,7b-octahydro-1,1,7,7a-tetramethyl-, [1aR-(1a.alpha.,7.alpha.,7a.alpha.,7b.alpha.)]-		2550(1431-3670)
Bicyclo[3.1.1]hept-2-ene-2-methanol, 6,6-dimethyl-		4607(0-4607)
Naphthalene, 1,2,3,4,4a,5,6,8a-octahydro-7-methyl-4-methylene-1-(1-methylethyl)-, (1.alpha.,4a.alpha.,8a.alpha.)-		2287(1241-3334)

Methyl Isobutyl Ketone		2207(1690-2724)
Anisole, p-allyl-		3896(0-3896)
1H-Benzocycloheptene, 2,4a,5,6,7,8-hexahydro-3,5,5,9-tetramethyl-, (R)-		1936(1143-2729)
Benzene, 1-methoxy-4-(1-propenyl)-		3570 (0-3570)
1,6,10-Dodecatriene, 7,11-dimethyl-3-methylene-, (E)-		1766 (951-2581)
3-Cyclopentene-1-acetaldehyde, 2,2,3-trimethyl-		1750 (932-2568)
Bicyclo[2.2.1]heptan-2-ol, 1,7,7-trimethyl-, (1S-endo)-		3168 (0-3168)
.alpha.-Phellandrene		1560 (1119-2001)
Cyclobuta[1,2:3,4]dicyclopentene, decahydro-3a-methyl-6-methylene-1-(1-methylethyl)-, [1S-(1.alpha.,3a.alpha.,3b.beta.,6a.beta.,6b.alpha.)		1395 (753-2038)
Eucalyptol		1317 (839-1796)

SUPPLEMENTARY DATA 2 (non-published material)

FOR (title):

Weathering of antibacterial melt-spun polyfilaments modified by pine rosin

by

Mikko Kanerva, Jacob Mensah-Attipoe, Arja Puolakka, T.M. Takala, Marko Hyttinen,

Rama Layek, Sarianna Palola, Vladimir Yudin, Pertti Pasanen, Per Saris

submitted 7.12.2020 (peer review)

Data description

This data is a detail report related to the VOC measurements described and analyzed in the paper **Weathering of antibacterial melt-spun polyfilaments modified by pine rosin.** The detailed description of the method, samples preparations and main outcomes are given in the manuscript. Figures (1-7) are the direct graphs from the device software as 'chromatograms' (TIC, total ion chromatograms) for each polyfilament fibre sample analyzed at different temperatures. The graphs indicate the measured (computed) ion count (abundance, arbitrary units) as a function of time (minutes).

a)
b)
c)

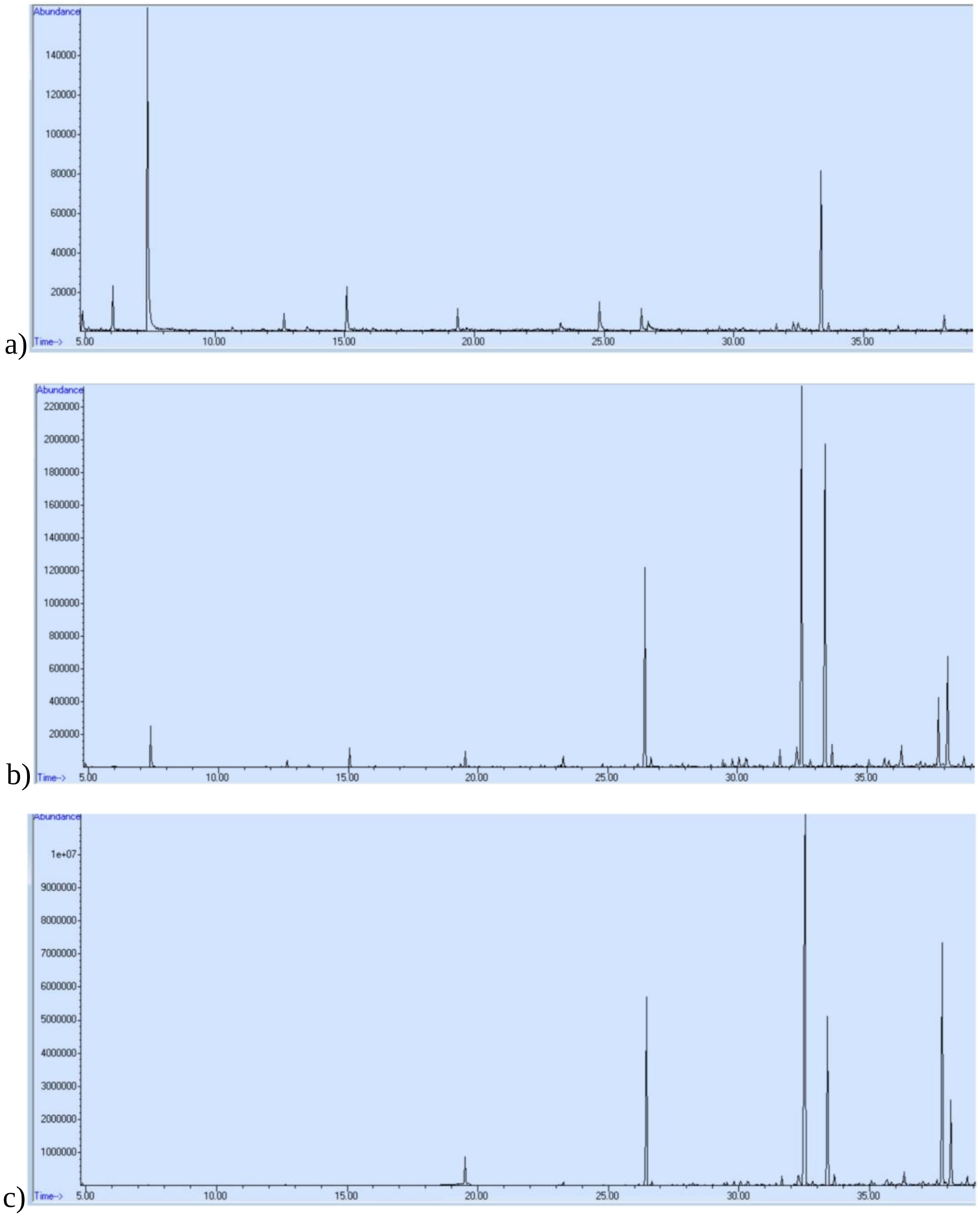

Figure 1. Measured emitted compounds from fPE analyzed at different temperatures: a) at 25 ºC; b) at 60 ºC; c) at 105 ºC. Note the different range of vertical axis for different temperature cases.

a) b) c)

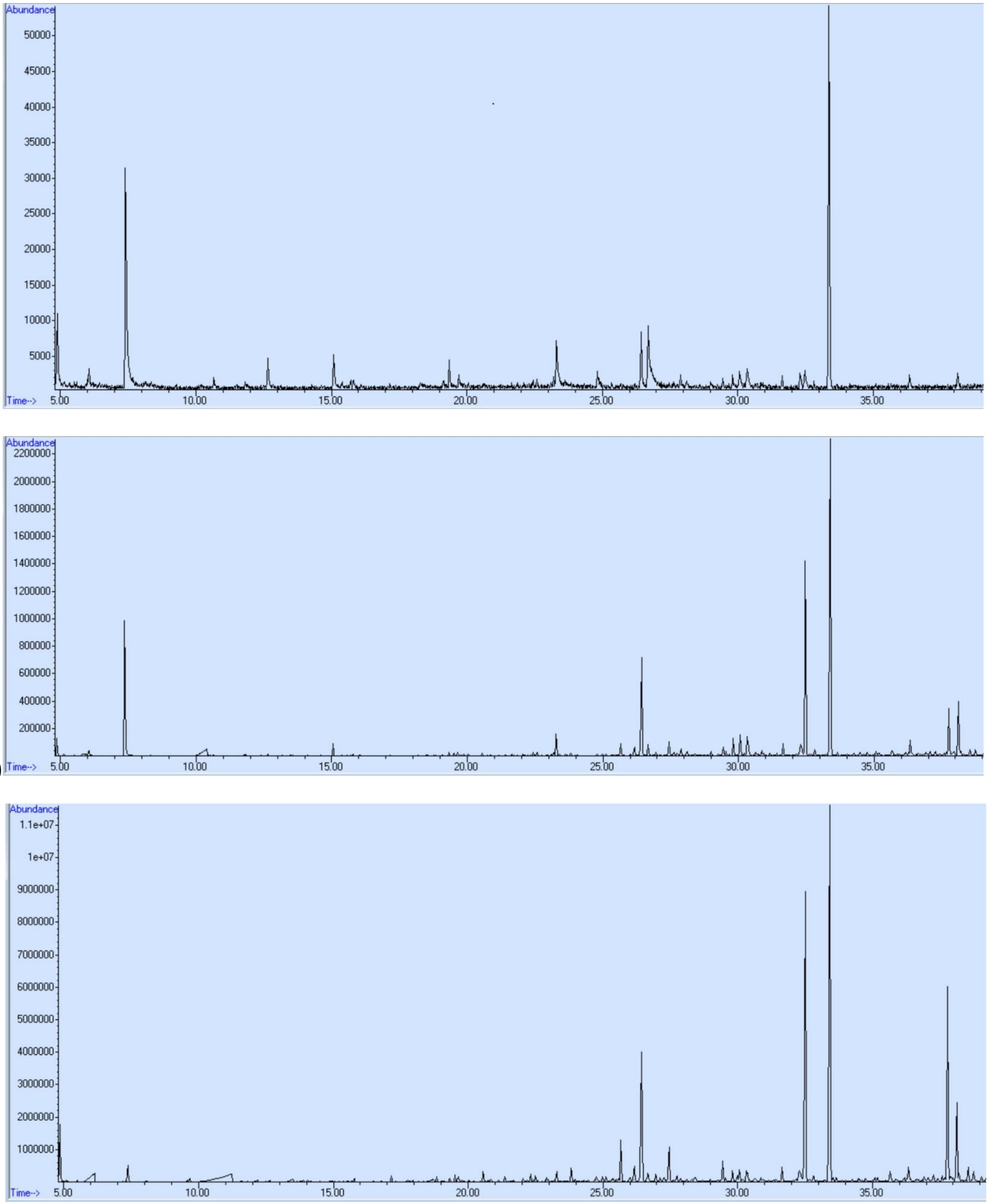

Figure 2. Measured emitted compounds from fPE10 analyzed at different temperatures: a) at 25 ºC; b) at 60 ºC; c) at 105 ºC. Note the different range of vertical axis for different temperature cases.

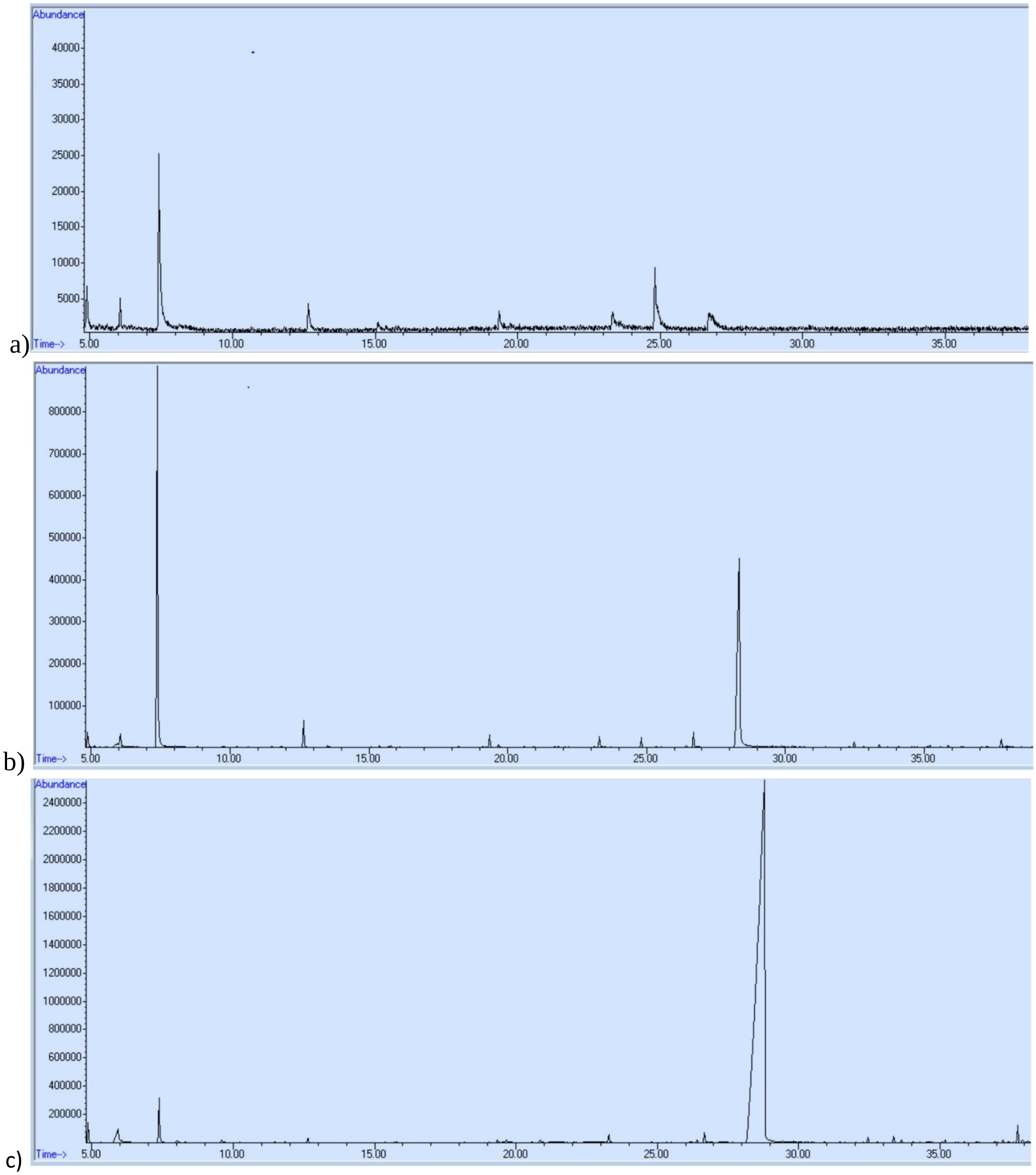

Figure 3. Measured emitted compounds from fPA analyzed at different temperatures: a) at 25 ºC; b) at 60 ºC; c) at 105 ºC. Note the different range of vertical axis for different temperature cases.

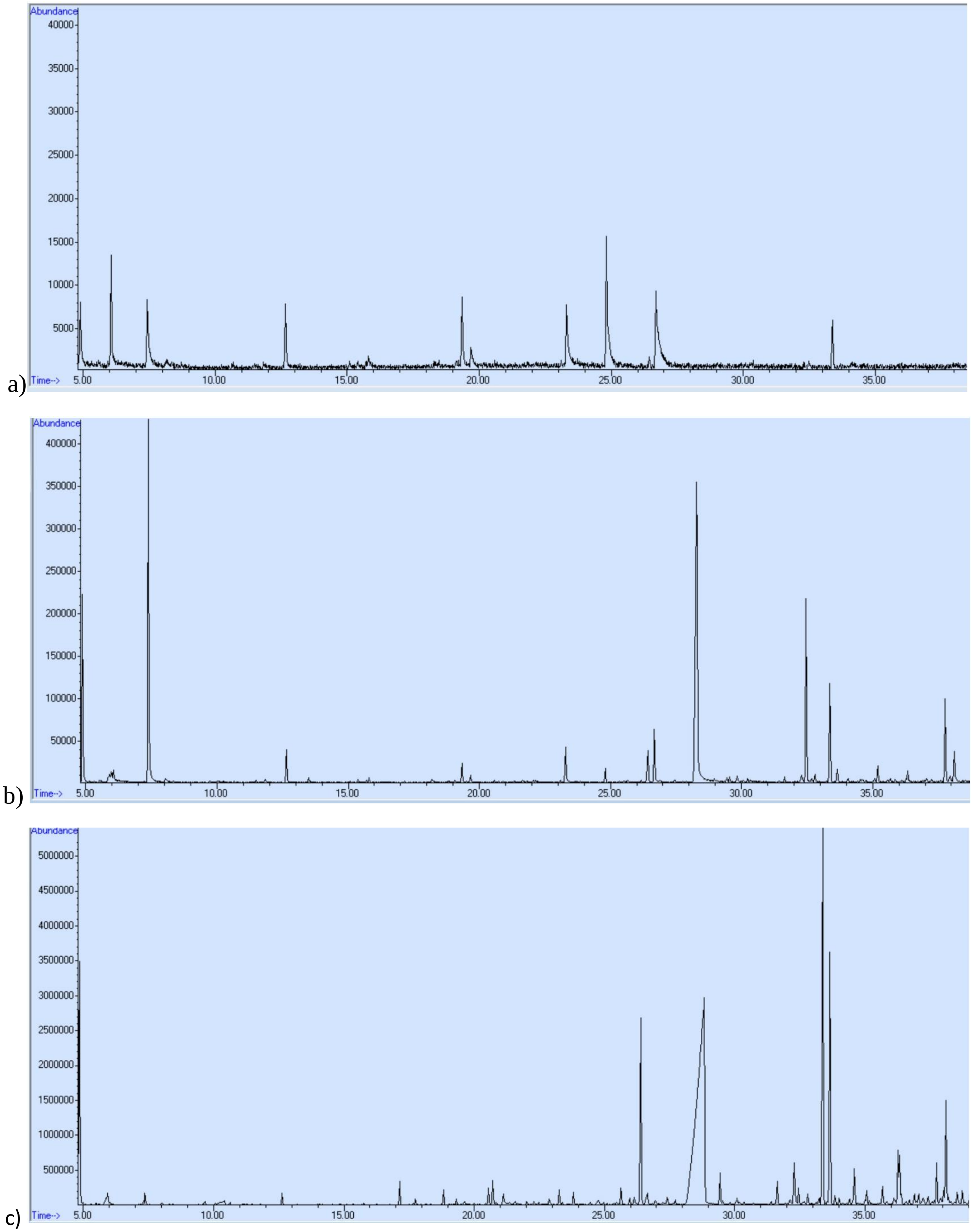

Figure 4. Measured emitted compounds from fPA10 analyzed at different temperatures: a) at 25 ºC; b) at 60 ºC; c) at 105 ºC. Note the different range of vertical axis for different temperature cases.

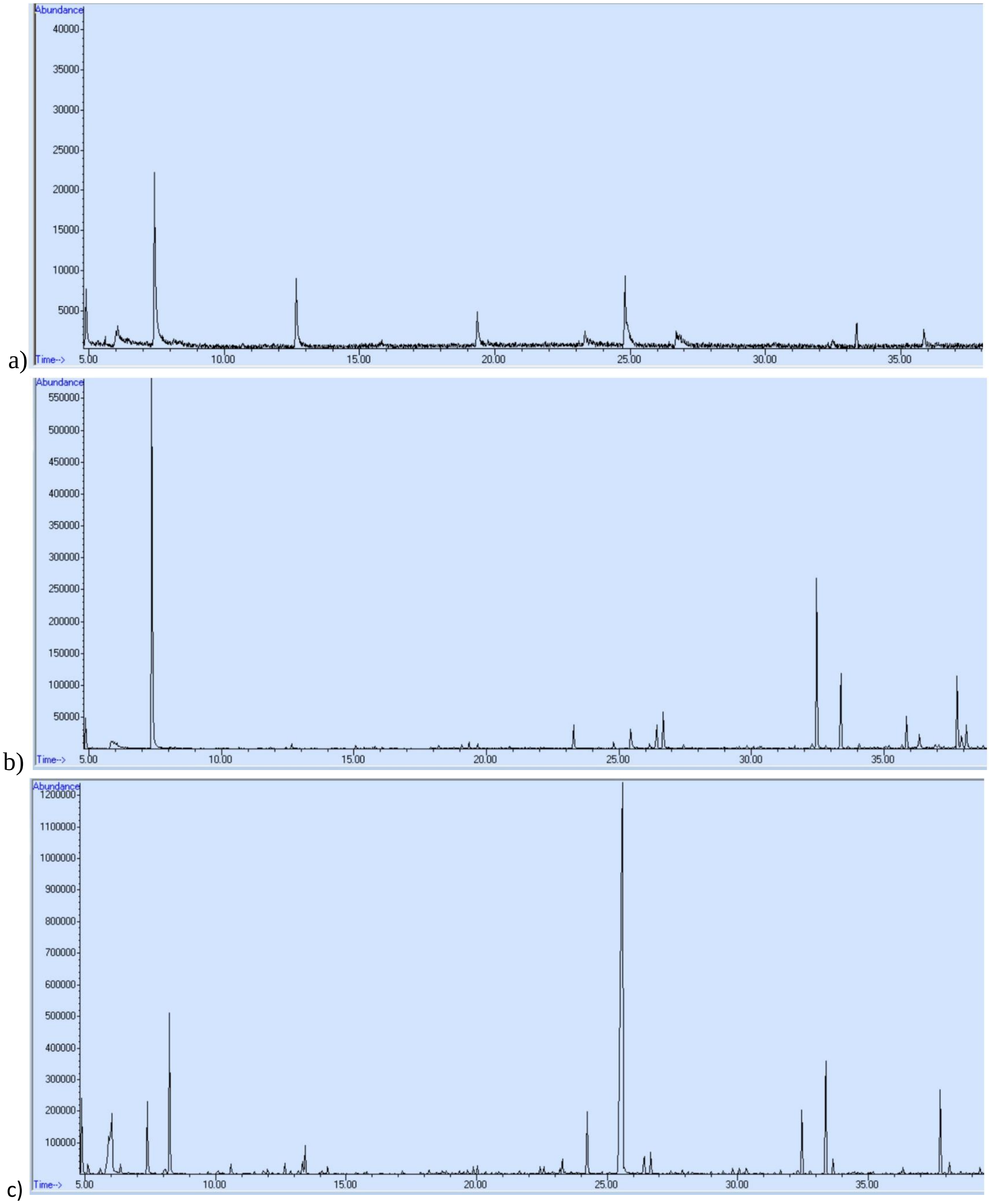

Figure 5. Measured emitted compounds from fPLA analyzed at different temperatures: a) at 25 ºC; b) at 60 ºC; c) at 105 ºC. Note the different range of vertical axis for different temperature cases.

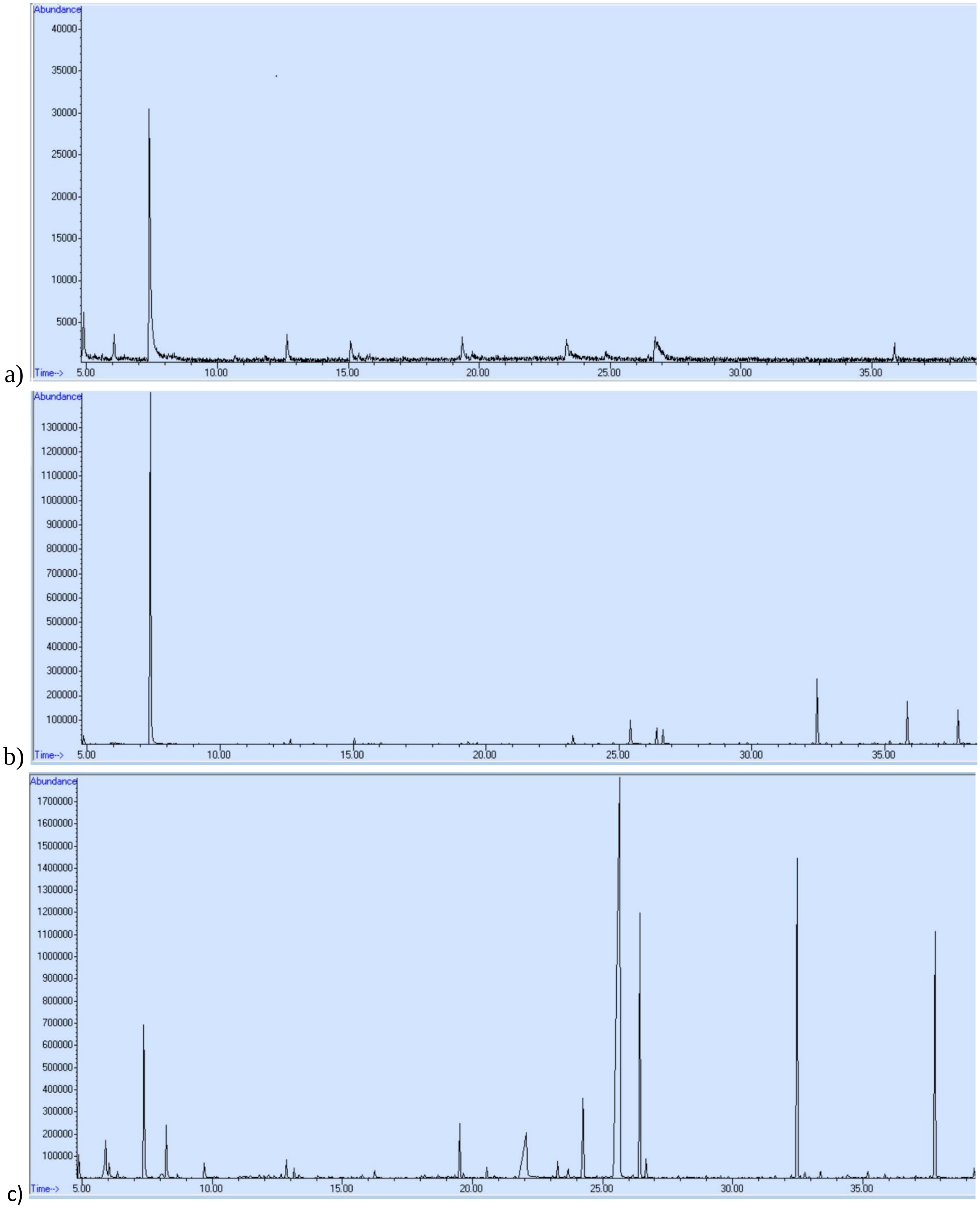

Figure 6. Measured emitted compounds from fPLA1Ag analyzed at different temperatures: a) at 25 ºC; b) at 60 ºC; c) at 105 ºC. Note the different range of vertical axis for different temperature cases.

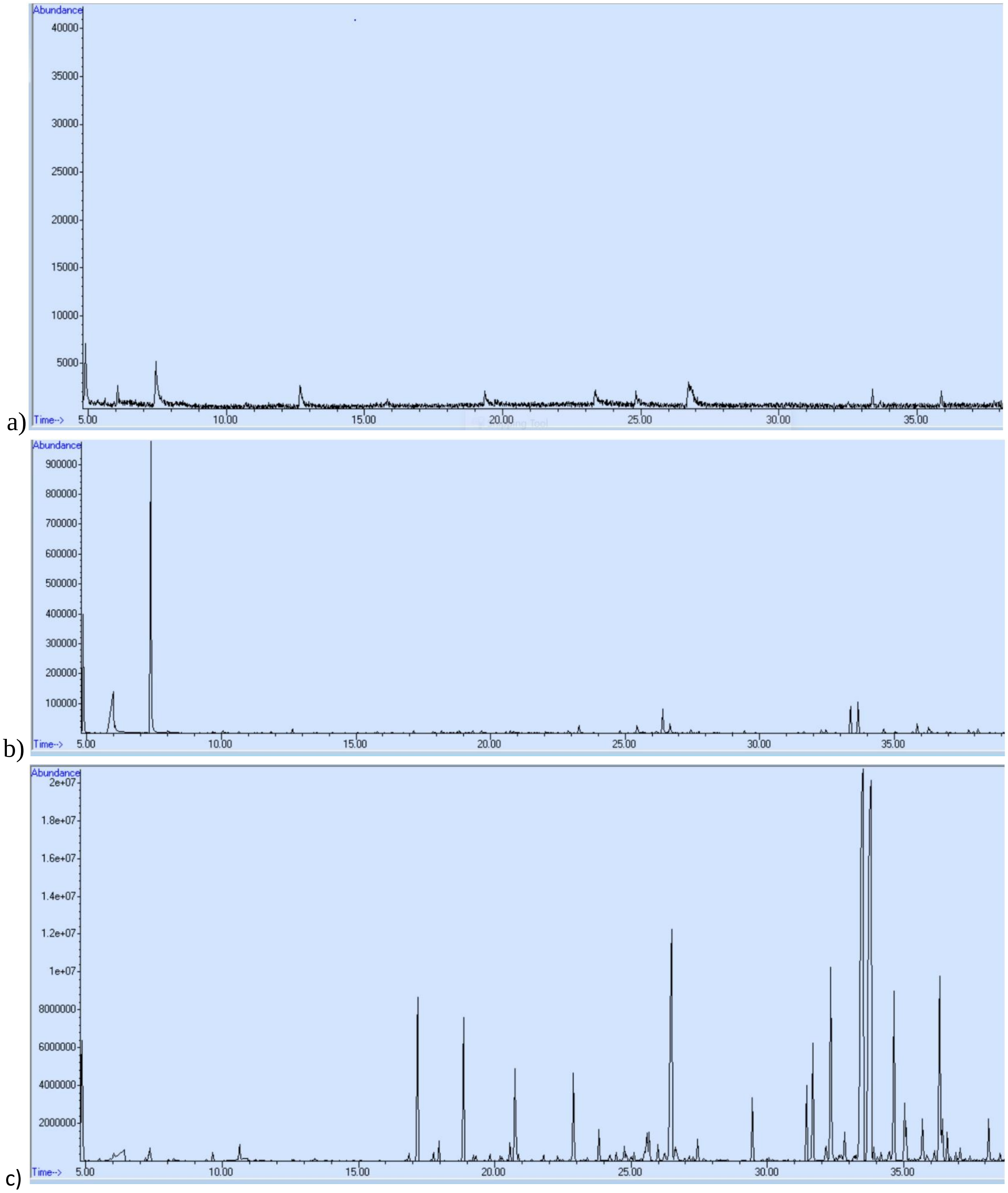

Figure 7. Measured emitted compounds from fPLA10 analyzed at different temperatures: a) at 25 ºC; b) at 60 ºC; c) at 105 ºC. Note the different range of vertical axis for different temperature cases.
